# Paediatric drug use with focus on *off-label* prescriptions in Swedish outpatient care – a nationwide study

**DOI:** 10.1111/j.1651-2227.2011.02287.x

**Published:** 2011-09

**Authors:** J Olsson, E Kimland, S Pettersson, V Odlind

**Affiliations:** 1 Medical Products AgencyUppsala, Sweden; 2 Department of Clinical Pharmacology, Karolinska University hospitalStockholm, Sweden

**Keywords:** Child, Drug prescription, Off-label, Primary care, Register study

## Abstract

**Aim:**

To determine drug prescription and proportion of off-label dispensing in the Swedish paediatric outpatient population.

**Methods:**

All dispensed outpatient prescriptions to children aged 0 < 18 years as well as the proportion of off-label drug use during 2007 were analysed using data from the Swedish Prescribed Drug Register.

**Results:**

In total, 2.19 million drug prescriptions of 898 different drug substances were dispensed to paediatric patients, and of those substances, 64% had been dispensed off-label at least once. The overall off-label rate of all prescriptions was 13.5%, among which topical drugs as well as sex hormones were the most commonly prescribed off-label drugs. More than half of all children in Sweden had received at least one prescribed drug in 2007.

**Conclusions:**

There is a high prescribing of medicines to children in outpatient care in Sweden with a considerable amount of off-label prescriptions. Topically administered drugs, sex hormones, antidepressants, hypnotics, cardiovascular drugs and nonsteroidal anti-inflammatory drugs were commonly prescribed off-label.

## Introduction

Until recently, clinical trials in children were often regarded as unethical. As a consequence, the majority of drugs prescribed to children have not been studied in the paediatric population. Hence, off-label prescribing is frequent in paediatric routine care (1–9). As children's growth and development might lead to different efficacy and/or safety patterns compared to the adult population, the need for studies in children is apparent.

A new legislation was introduced in 2006 to improve the availability of safe and effective medicines for children in Europe (10). According to the new legislation, the development and accessibility of effective and safe medicinal products for paediatric patients should be facilitated by identifying therapeutic areas in need of further research. For this purpose, an inventory of current prescribing patterns to children was requested from all EU Member States.

The objectives of this study were to investigate the drug prescriptions in the Swedish paediatric outpatient population and to analyse the proportion of off-label drug use.

**Key notes**

- This study indicates that a high proportion of Swedish children are prescribed drugs in out-patient care and that there is a considerable off-label drug prescribing. To improve effective and safe use of drugs in children, more paediatric research is needed, in particular for topically administered drugs, cardiovascular drugs, antidepressants, hypnotics and several NSAIDs.

## Methods

This is a nationwide study that includes all drug prescriptions to children aged 0 < 18 years in Sweden during 2007. Data were received as dispensed drug prescriptions at pharmacies from the Swedish Prescribed Drug Register (SPDR) hosted by the Centre of Epidemiology at the National Board of Health and Welfare. The data set included 2.19 million drug prescriptions of a total of 898 different drug substances that had been dispensed to 968 465 paediatric patients between 0 < 18 years. Of the 898 different substances, 386 were dispensed more than 100 times and those comprised 99.6% of the overall number of dispensations for children in the SPDR and were included in the off-label analysis. Available information comprised product identification, patient's date of birth and date of purchase. There was no information with regard to dose or indication available in the SPDR at the time of data collection. The drugs were categorized according to the Anatomical Therapeutic Chemical (ATC) classification on the fifth level and analysed regarding number of dispensed drug prescriptions (11).

Data regarding the population were received from Statistics Sweden (12).

Distribution of dispensed drug prescriptions was divided into six different age groups: 0 < 1, 1 < 2, 2 < 6, 6 < 12, 12 < 16 and 16 < 18 years. This age classification was based on the patient's age at the time of drug prescribing.

### Off-label classification

The licence status of each drug was determined according to the Summary of Product Characteristics (SmPC) (13,14). In particular, information in sections 4.1, 4.2, 4.3 and 4.4, concerning indications, posology, contraindications and warnings and precautions, was used. Secondary reference source for assessing licensed status was the published booklet containing details about pharmacy prepared drugs for children (15).

A dispensed drug prescription was regarded as off-label if it was given outside the terms of the drug labelling, based on four different categories:

1. A drug substance dispensed outside the age-limit in the SmPC
2. A substance that lacked paediatric information in the SmPC
3. A substance for which the SmPC stated that paediatric clinical studies had not been performed
4. A substance that was explicitly not recommended or contraindicated in children

Moreover, all unauthorized substances were coded as off-label. Also, all pharmacy prepared drugs were coded as off-label unless they were mentioned in the second reference source (15).

For practical reasons, the off-label classification was based on substance instead of the specific dispensed medicinal product. Therefore, it was necessary to make an assumption that the product dispensed represented the widest on-label age interval for that substance.

One substance could be classified off-label in more than one of the four categories. For seven substances, an age-limit could not be identified, and therefore, they were excluded from further analysis.

## Results

Half (51%) of all children in Sweden had received at least one prescribed drug during 2007 with a considerably higher rate among children below age two (Table 1). The most commonly prescribed drugs were drugs for the respiratory tract (ATC-code R) and antibiotics (ATC-code J; Table 2).

**Table 1:** Total paediatric population 2007; number of unique individuals with prescriptions; percentage of all children with at least one prescribed drug; number of dispensed prescriptions; dispensed prescriptions per patient, all by age groups

Age groups (years)	Total population N	Number of unique individuals N	Percentage with prescribed drug %	Number of dispensed prescriptions N	Dispensed prescriptions/patient
0 < 1	92 064	66 203	72	145 878	2.20
1 < 2	107 351	79 003	74	207 718	2.63
2 < 6	406 797	235 629	58	537 537	2.28
6 < 12	570 620	243 690	43	502 982	2.06
12 < 16	472 522	200 216	42	445 145	2.22
16 < 18	262 063	143 724	55	358 053	2.49
All ages	1 911 417	968 465	51	2 197 313	2.27

**Table 2:** Total number of dispensed substances within each anatomical therapeutic chemical (ATC)-group and the percentage of dispensed drug prescriptions classified as off-label

ATC- codes	Dispensations N	All off-label categories %
All	2 177 859	13.5
A. Alimentary tract & metabolism	93 745	11.1
B. Blood & blood forming organs	12 314	9.3
C. Cardiovascular system	14 654	18.3
D. Dermatologicals	196 333	29.5
G. Genitourinary system & sex hormones	95 241	97.3
H. Systemic hormonal preparations excluding sex hormones and insulins	49 685	1.0
J. Anti-infectives for systemic use	703 177	0.1
L. Antineoplastic & immune-modulating agents	1326	0.8
M. Musculo-skeletal system	44 360	9.6
N. Nervous system	94 265	12.8
P. Antiparasitic products, insecticides & repellents	19 243	0.3
R. Respiratory system	678 823	3.1
S. Sensory organs	173 436	52.4
V. Various	1257	0

Two hundred and forty-seven (64%) of the 386 substances included in the off-label analysis had been prescribed off-label at least once, resulting in 295 000 off-label drug prescriptions, constituting 13.5% of all dispensed prescriptions to children. Pharmacy prepared drugs or unauthorized drugs accounted for 1.6% of all dispensed drugs.

In total, 512 substances were excluded from analysis as they had been prescribed <100 times during 2007. This scattered group constituted around 0.4% of all dispensations. A preliminary analysis of those 512 excluded substances showed that at least 150 of them were assessed as on-label according to the criteria for this study.

The distribution of dispensed substances and the proportion of off-label classification by ATC code are presented in Table 2. Drugs for infectious and respiratory tract diseases were the most commonly dispensed substances, but had a low off-label proportion. The highest off-label proportion was found among drugs for topical use (for example topical antifungals, fusidic acid, steroids and isotretinoin) and for sex hormones (mainly hormonal contraceptives). Other areas with a clinically important proportion of off-label drug use were found among cardiovascular and psychiatric (antidepressants, hypnotics, e.g. melatonin) drugs as well as among drugs for the musculoskeletal system (mainly nonsteroidal anti-inflammatory drugs - NSAIDs).

The most common reason for off-label classification in general was a lack of paediatric information in the SmPC as shown in Table 3. For example, with regard to cardiovascular drugs as well as to sex hormones, there was sparse or nearly complete absence of paediatric information in the SmPC, resulting in off-label classification. In contrast, dispensed drugs for the musculoskeletal system and psychiatric drugs were mainly classified as off-label because of age or stated lack of paediatric studies, or the drug was explicitly not recommended or contraindicated in children.

**Table 3:** Distribution of reasons for off-label classification of all dispensed drugs classified as off-label

ATC-code	Off-label N	Age (i), Lack of paediatric clinical studies (iii), Not recommended/contraindicated in children (iv)	Absence of paediatric information (ii)	Unauthorized drugs and pharmacy prepared drugs
All	294 627	18.8	74.5	6.7
A	10 452	29.9	69.8	0.3
B	1145	43.2	56.8	0
C	2687	0	100	0
D	57 915	19.2	53.2	27.6
G	92 693	9.0	91.0	0
H	501	0	100	0
J	898	100	0	0
L	11	100	0	0
M	4274	97.5	2.5	0
N	12 043	76.4	20.5	3.1
P	63	100	0	0
R	21 137	72.0	15.1	12.9
S	90 808	3.0	96.5	0.5
V	0	0	0	0

ATC, anatomical therapeutic chemical.

## Discussion

In accordance with previous studies, high paediatric use of antibiotics and of drugs for the respiratory tract was found, mostly considered to be on-label with regard to age and product information (5,8,16).

In a US population-based study, around 56% of children <12 had used at least one medicinal product during the week preceding the study (17). Whether our finding that more than 70% of young children had been prescribed a medicine in 2007 is in accordance with the US finding is not possible to assess because of different methodology.

The overall off-label rate of 13.5% found in this study was slightly lower compared to a previous Swedish study in paediatric outpatients in the Stockholm area, reporting an off-label rate of 20.7% (4). This difference can be explained by fewer off-label categories and analysis of substances instead of medicinal products in the present study (4). Still, the magnitude of off-label prescription is similar to that reported in other studies (4,6,8).

The off-label proportion may have been underestimated as the present study was restricted to the most commonly dispensed drugs, although they represented more than 99% of all dispensed drugs. The excluded drugs probably comprised drugs dispensed for children with rare diseases. It is likely that several of those drugs had been prescribed off-label. However, even if all those rarely prescribed drugs had been classified as off-label, the off-label rate in the study would not have changed much. The lack of possibility to assess additional off-label categories such as dose and indication could also have resulted in an underestimation of the off-label proportion.

As data were retrieved from SPDR at the fifth ATC level, no off-label analysis could be performed regarding the formulation of the medicinal product, which also could have contributed to an underestimation of the off-label proportion.

Topically used drugs and sex hormones were associated with a high off-label proportion as also shown elsewhere (8). However, sex hormones, mostly hormonal contraceptives, are perhaps not the most important in need of further paediatric research, because long clinical experience does not suggest different efficacy in the paediatric population. However, there is still a need for long-term safety follow-up. Topically used drugs, antidepressant drugs, hypnotics, cardiovascular drugs and different NSAIDs are considered in greater need of paediatric clinical studies as they represent important therapeutic areas in all paediatric age groups with very different dose requirement as also suggested by other investigators (8). Antidepressive agents have been highlighted in adolescents as drugs with a different adverse drug reaction pattern compared to that seen in adults, supporting the need for more paediatric clinical research both on drug efficacy and on safety (18,19).

As the present study, other studies on outpatient paediatric drug use have demonstrated that the most common reason for off-label classification is complete lack of paediatric information in the SmPC (4,20). A similar finding was also reported in a recent prospective hospital-based study in Sweden (1).

## Conclusion

There is a high prescribing of medicines to children in outpatient care in Sweden. This population-based register study indicates, in congruence with similar studies, that there is a considerable off-label drug prescribing to children in outpatient care in Sweden. The study identified some important areas, such as topically administered drugs, sex hormones, cardiovascular drugs, antidepressants, hypnotics and several NSAIDs, where there appears to be a need for more paediatric research to improve safe and effective use of drugs in children.
